# A new twist on an old idea: a two‐dimensional speckle tracking assessment of cyclosporine as a therapeutic alternative for heart failure with preserved ejection fraction

**DOI:** 10.1002/phy2.174

**Published:** 2013-12-05

**Authors:** Jessica A. Hiemstra, Songtao Liu, Mark A. Ahlman, Karl H. Schuleri, Albert C. Lardo, Christopher P. Baines, Kevin C. Dellsperger, David A. Bluemke, Craig A. Emter

**Affiliations:** 1 Department of Biomedical Science, University of Missouri‐ Columbia, Columbia, Missouri; 2 Radiology and Imaging Sciences, National Institutes of Health Clinical Center, Bethesda, Maryland; 3 Molecular Biomedical Imaging Laboratory, National Institute of Biomedical Imaging and Bioengineering, Bethesda, Maryland; 4 Division of Cardiology, Department of Medicine, Johns Hopkins University, Baltimore, Maryland; 5 Dalton Cardiovascular Research Center, University of Missouri‐ Columbia, Columbia, Missouri; 6 Department of Medical Pharmacology and Physiology, University of Missouri‐ Columbia, Columbia, Missouri; 7 Department of Internal Medicine, University of Missouri‐ Columbia, Columbia, Missouri; 8 Center for Health Care Quality, University of Missouri‐ Columbia, 1600 E. RollinsW160 Veterinary Medicine, Columbia, 65211, Missouri

**Keywords:** 2D speckle tracking, CT, cyclosporine, diastolic heart failure, HFpEF

## Abstract

We recently reported that mitochondrial dysfunction, characterized by increased mitochondrial permeability transition (MPT), was present in a translational swine model of heart failure with preserved ejection fraction (HFpEF). Cyclophilin D is a key component of the MPT pore, therefore, the purpose of this study was to test the efficacy of a novel cyclosporine (CsA) dosing scheme as a therapeutic alternative for HFpEF. Computed tomography (CT), two‐dimensional speckle tracking two‐dimensional speckle tracking (2DST), and invasive hemodynamics were used to evaluate cardiac function. CT imaging showed 14 weeks of CsA treatment caused eccentric myocardial remodeling (contrasting concentric remodeling in untreated HF animals) and elevated systemic pressures. 2DST detected left ventricular (LV) mechanics associated with systolic and diastolic dysfunction prior to the onset of significantly increased LV end diastolic pressure including: (1) decreased systolic apical rotation rate, longitudinal displacement, and longitudinal/radial/circumferential strain; (2) decreased early diastolic untwisting and longitudinal strain rate; and (3) increased late diastolic radial/circumferential mitral strain rate. LV mechanics associated with systolic and diastolic impairment was enhanced to a greater extent than seen in untreated HF animals following CsA treatment. In conclusion, CsA treatment accelerated the development of heart failure, including dilatory LV remodeling and impaired systolic and diastolic mechanics. Although our findings do not support CsA as a viable therapy for HFpEF, 2DST was effective in differentiating between progressive gradations of developing HF and detecting diastolic impairment prior to the development of overt diastolic dysfunction.

## Abstract (precis)

We recently reported that mitochondrial dysfunction, characterized by increased mitochondrial permeability transition (MPT), was present in a translational swine model of heart failure with preserved ejection fraction (HFpEF). Cyclophilin D is a key component of the MPT pore, therefore, the purpose of this study was to test the efficacy of a novel cyclosporine (CsA) dosing scheme as a therapeutic alternative for HFpEF. CsA treatment accelerated the development of heart failure, including dilatory LV remodeling and impaired systolic and diastolic mechanics. Although our findings do not support CsA as a viable therapy for HFpEF, 2DST was effective in differentiating between progressive gradations of developing HF and detecting diastolic impairment prior to the development of overt diastolic dysfunction.

## Introduction

Although heart failure with preserved ejection fraction (HFpEF) has been clinically recognized for over three decades, it remains somewhat of an enigma. It is known HFpEF patients are a heterogeneous group displaying multiple pathological mechanisms and comorbidities such as hypertension, obesity, diabetes, chronic kidney disease, and aging (Maeder and Kaye 2009; Borlaug and Paulus 2011). Patients with HFpEF are largely unresponsive to current therapies proven effective in heart failure patients with reduced systolic function (HFrEF), suggesting these heart failure subtypes are distinct from one another with divergent pathophysiology (Maeder and Kaye 2009; Paulus and van Ballegoij 2010; Borlaug and Paulus 2011; Borlaug and Redfield 2011; Burkhoff 2012; Zile et al. 2013). Furthermore, HFpEF is difficult to diagnose given the compensated state of resting cardiac function, often requiring an additional physiological stressor such as exercise testing in order to reveal myocardial dysfunction (Borlaug et al. 2006, 2010a,b; Ennezat et al. 2008; Tan et al. 2009; Maeder et al. 2010; Haykowsky et al. 2011). As a result, there is a critical need for research examining novel treatment options for HFpEF patients and identification of effective noninvasive mechanisms of monitoring cardiac function (Heidenreich et al. 2013).

The link between impaired myocardial energetics, mitochondrial dysfunction, and cell death is well established in heart failure (Zhang 2002; Ventura‐Clapier et al. 2004; Foo et al. 2005; Dorn 2009). Previous work from our laboratory demonstrated the presence of mitochondrial dysfunction, specifically increased susceptibility to mitochondrial permeability transition (MPT), in a translational swine model of HFpEF (Emter and Baines 2010). This energetic impairment may be linked to our recent report of increased isovolumic relaxation (Tau), an energy‐dependent phase of relaxation, in the same model (Marshall et al. 2013). This study was designed to examine the physiological impact of inhibiting cyclophilin D, a primary molecular component of the MPT pore, on cardiac function using cyclosporine (CsA). Immunosuppressive doses of CsA were thoroughly examined as inhibitors of calcineurin and cardiac remodeling in heart failure (Frey and Olson 2003), and an early clinical trial for ischemia‐reperfusion/myocardial infarction treatment, which has now moved into phase III (The CIRCUS trial), showed CsA limits reperfusion injury following acute myocardial infarction (Piot et al. 2008). In contrast to past studies, we used a reduced nonimmunosuppressive dose, which would prevent MPT via inhibition of cyclophilin D without subsequent interference of calcineurin signaling or associated cardiac remodeling (Okumi et al. 2008; Piot et al. 2008; Rigol et al. 2008; Marechal et al. 2011). We hypothesized CsA‐dependent inhibition of MPT would attenuate the diastolic dysfunction previously observed and ultimately, the development of HFpEF via improved myocardial energetics. To test this hypothesis, we used 2D speckle tracking echocardiography, a sensitive noninvasive technique of evaluating myocardial mechanics that has recently gained attention as an effective means of diagnosing HFpEF (Kosmala et al. 2008; Norman et al. 2011; Yip et al. 2011; Morris et al. 2012a). The purpose of this study was to test the efficacy of a novel CsA dosing scheme as a viable therapeutic alternative for HFpEF. For this study, we used a translational and clinically relevant mini‐swine model of HFpEF that exhibits key myocardial pathophysiological characteristics of the disease, including diastolic dysfunction, depressed contractile reserve, fibrosis, hypertrophy, and increased natriuretic peptide expression (Marshall et al. 2013) providing an ideal setting for the study of novel therapeutic approaches in this population.

## Methods

### Aortic banding and cyclosporine treatment

Before aortic banding, intact male Yucatan miniature swine (27–30 kg; 8 months old) were matched for body mass and cardiac function then assigned into three groups: nonsham sedentary control (CON; *n* = 5), banded HF sedentary (HF; *n* = 5), and banded HF CsA treated (HF‐CsA; *n* = 5). Heart failure was induced by aortic banding for a period of 20 weeks using methods previously published by our laboratory (Marshall et al. 2013). A systolic transstenotic gradient of ~70 mmHg (73 ± 2, 74 ± 1, for HF and HF‐CsA, respectively, *P* = NS) was achieved while maintaining a distal peripheral vascular mean arterial pressure (MAP) of ~90 mm Hg (93 ± 1, 90 ± 1, for HF and HF‐CsA, respectively, *P* = NS) under anesthesia using phenylephrine (I.V. 1–3 μg kg^−1^ min^−1^) at a heart rate of 100 beats/min (100 ± 5, 107 ± 2, for HF and HF‐CsA, respectively, *P* = NS). Following the development of left ventricular (LV) hypertrophy, treatment with CsA (2.0 mg kg^−1^day^−1^, oral) or placebo began 6 weeks post aortic banding and continued daily for 14 weeks. Animals were fed a standard diet averaging 15–20 g/kg once daily, and water was provided ad libitum. Dissection of vital tissues occurred at the time of death. All animal protocols were in accordance with the “Principles for the Utilization and Care of Vertebrate Animals Used in Testing Research and Training” and approved by the University of Missouri Animal Care and Use Committee.

### In vivo cardiovascular function

Central and peripheral hemodynamic measures were collected 20 weeks post aortic banding as described previously (Marshall et al. 2013). Animals were initially anesthetized with a telazol (5 mg/kg)/xylazine (2.25 mg/kg) mix and maintained on propofol (6–10 mg kg^−1^ min^−1^ with bolus as needed). Heparin was given with an initial loading dose of 300 U/kg i. v., followed by maintenance of 100 U/kg each hour. A median sternotomy was performed and the pericardium opened at the apex for insertion of catheters. Great care was taken to leave the pericardium as intact as possible. A custom fluid‐filled angiocatheter was inserted into the apex of the heart for measurement of LV pressure, advanced into the aorta for measurement of peripheral systemic MAP in the aorta (distal to the aortic band in HF groups), and data were recorded using LabChart (ADInstruments, Inc., Colorado Springs, CO). Animals were allowed to stabilize for 10 min after LV catheter placement until a stable pressure and heart rate pattern were observed. This state of homeostasis was labeled “Resting”. Catheter placement was visualized and confirmed using angiography (Infimed software, Palo Alto, CA).

### Computed tomography imaging

CT image collection, reconstruction, and analysis were performed as previously described (Bluemke et al. 2008; Chen et al. 2013). Animals were scanned with electrocardiographic (ECG) monitoring using a second‐generation 320 detector row CT unit (Aquilion ONE ViSION; Toshiba Medical Systems, Otawara, Japan). A 60 mL bolus of iodixanol (Visipaque 320 mg iodine/mL, GE Healthcare, Oslo, Norway) was injected intravenously at rate of 5 mL/sec, opacifying the LV chamber during first pass (Bluemke et al. 2008). During CT acquisition, respiration was suspended and imaging performed using a retrospectively gated protocol with the following parameters: three R‐R intervals, gantry rotation time 275 msec, detector collimation 0.5 mm × 320, tube voltage 120 kV, and tube current 700 mA (Chen et al. 2013). From every 5% of the R‐R interval, raw data were reconstructed to form an isotropic 512 × 512 matrix with contiguous 0.5 mm slice thickness. Multiple segment iterative reconstruction algorithms (AIDR3D standard; Toshiba Medical Systems), and a standard soft tissue kernel (FC03) were used. Temporal resolution based on gantry rotation of multidetector CT acquisitions was 45.8 msec. Cardiac EF% and size (LV and atrial systolic/diastolic volumes) were measured using Vitrea workstation (Vitrea fx 6.3; Vital Images, Minnetonka, MN).

### Two‐dimensional speckle tracking echocardiography

Transthoracic echocardiography was performed under inhaled isoflurane anesthesia (0.5%) in the supine/right lateral position 2 and 14 weeks after beginning treatment with CsA using a GE Vivid I Ultrasound system as previously described by our lab (Emter and Baines 2010; Marshall et al. 2013). Analysis was performed offline using GE EchoPac Software. LV end diastolic dimension and wall thickness were measured using M‐mode recordings (Emter and Baines 2010; Marshall et al. 2013). Six segments of the LV and septum were generated from apical four‐chamber and short‐axis two‐dimensional views (acquired at the mitral‐valve and apex levels) and averaged to determine global strain, strain rate, and displacement in the longitudinal, transverse, radial, and circumferential dimensions over three cardiac cycles (Mondillo et al. 2011; Marshall et al. 2013). Torsion was calculated as the difference between mitral and apical end systolic rotation (degrees) and normalized to both LV hypertrophy (wall thickness) and end diastolic chamber length as previously described (Russel et al. 2009; Marshall et al. 2013).

### Statistical analysis

All data analysis was performed using SPSS version 19.0 (IBM Corporation, Armonk, NY) or SigmaStat version 3.5. Group comparisons were made using either one‐way or repeated measures analysis of variance (ANOVA). Group differences revealed by ANOVA were found using Student Newman‐Keuls post hoc analysis. Within group comparisons were made using paired samples *t*‐test. All data are means ± SE, and significance is reported at *P* < 0.10 and *P* < 0.05 levels (Williams et al. 1997; Curran‐Everett and Benos 2004; Emter et al. 2005, 2011; Emter and Baines 2010; Marshall et al. 2013).

## Results

### LV Remodeling

Myocardial hypertrophy occurred in all aortic‐banded groups regardless of drug intervention. After 1 month, aortic banding significantly increased LV diastolic wall thickness by 18% in HF (*P* < 0.05, 5.5 ± 0.3 and 6.7 ± 0.4 mm for baseline preaortic banding and 1 month post banding, respectively; paired‐samples *t*‐test) but not CON animals (*P* = NS, 5.2 ± 0.3 and 5.9 ±0.3 mm for baseline preaortic banding and 1 month post banding, respectively; paired‐samples *t*‐test) and was significantly different between groups 2 months post aortic banding (*P* < 0.05; 5.6 ± 0.5 and 7.5 ± 0.3 mm for CON and HF, respectively) similar to previous findings from our lab (Marshall et al. 2013). LV end diastolic dimension was not altered 1 month post aortic banding (*P* = NS; 44.1 ± 1.1 and 42.2 ± 0.8 mm for CON and HF, respectively). In total, these results indicate concentric LV hypertrophy (an observation commonly associated with LV pressure overload) was present prior to the onset of CsA treatment. No differences in echocardiographic measures of morphology existed between HF and HF‐CsA groups at this time, therefore, data from both aortic‐banded groups were combined prior to the start of CsA treatment. CT assessment of LV morphology (Table 1) indicated LV mass was increased in the HF‐CsA group compared to CON and HF. LV end systolic and diastolic volumes (LV EDV and LV ESV, respectively) were significantly increased in the HF‐CsA group compared to CON and HF animals, as were left atrial (LA) volumes. When normalized to LV EDV, LV mass was increased only in the HF group. These findings indicate that although LV remodeling occurred in both HF groups, the hypertrophy was concentric in HF animals as opposed to eccentric (i.e., dilated) in the HF‐CsA group.

**Table 1.** CT and Hemodynamic analysis of resting systolic and diastolic function 20 weeks post aortic banding.

	CON	HF	HF‐CsA
Systolic function			
HR (beats/min)	126 ± 5	106 ± 4^*^	114 ± 4^§^
MAP (mmHg)	53 ± 11	59 ± 4	86 ± 8^*^^,^^†^
LV ESV (mL)	32 ± 3	24 ± 2	43 ± 3^†^
LV ESP (mmHg)	65 ± 7	81 ± 6	109 ± 9^*^
LV EF (%)	54 ± 2	56 ± 4	47 ± 3
LA ESV (mL)	25 ± 2	28 ± 1	44 ± 3^*^^,^^†^
LA EF (%)	47 ± 1	40 ± 5	33 ± 2^*^
Diastolic function			
LV EDV (mL)	70 ± 8	57 ± 6	80 ± 4^†^
LV EDP (mmHg)	11 ± 1	11 ± 1	15 ± 1^*^^,^^†^
LA EDV (mL)	47 ± 2	46 ± 1	65 ± 3^*^^,^^†^
Morphology			
LV Mass (g)	85 ± 4	87 ± 3	103 ± 3^*^^,^^†^
LV Mass:Vol (g/mL)	1.2 ± 0.1	1.7 ± 0.1^*^^,^^‡^	1.3 ± 0.1

Values are means ± SE. HR, heart rate; MAP, mean arterial pressure; LV ESV, left ventricular end systolic volume; LV ESP, left ventricular end systolic pressure; LV EF, left ventricular ejection fraction; LA ESV, left atrial end systolic volume; LA EF, left atrial ejection fraction; LV EDV_,_, left ventricular end diastolic volume; LV EDP_,_, left ventricular end diastolic pressure; LA EDV, left atrial end diastolic volume; LV mass:Vol, LV free wall mass:LV EDV ratio. Significance is indicated at *P < 0.05 versus CON; †*P* < 0.05 versus HF; ‡*P* < 0.05 versus HF‐CsA; §*P* = 0.08 versus CON.

**Figure 1.**
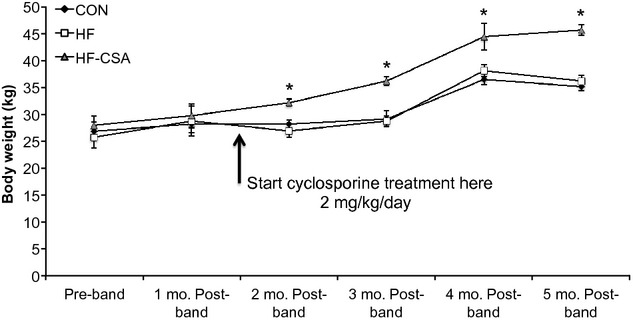
Cyclosporine treatment increases body weight (*P < 0.05 HF‐CsA versus CON and HF).

Postmortem assessment of LV morphology supports our CT imaging data. Body weight was significantly increased in the HF‐CsA group (Fig. 1, Table 2), therefore, heart morphology measures are reported both absolute and relative to body weight. Absolute whole heart weight was significantly increased in both HF and HF‐CsA groups compared to CON. However, when normalized to body weight significant hypertrophic remodeling occurred only in HF animals illustrating the coherence of our gross and imaging data.

**Table 2.** Group weight and postmortem assessment of whole heart morphology.

	CON	HF	HF‐CsA
Body weight (kg)	35 ± 1	36 ± 1	46 ± 1^*^^,^^†^
Heart weight (g)	174 ± 6	206 ± 11^*^	245 ± 4^*^^,^^†^
HW:BW (g/kg)	5.0 ± 0.2	5.7 ± 0.3^‡^	5.4 ± 0.1

Values are means ± SE. HW:BW, heart weight:body weight ratio. Significance is indicated at *P < 0.05 versus CON; †*P* < 0.05 versus HF; ‡*P* < 0.10 versus CON.

### LV function and hemodynamics

Resting hemodynamic and LV functional data 20 weeks post aortic banding are presented in Table 1. In general, systolic function was depressed in HF‐CsA animals compared to CON and HF groups. In HF‐CsA animals LV EF was reduced (47 ± 3%) compared to CON and HF groups (54 ± 2 and 56 ± 4, respectively; *P* = 0.108; Table 1). Additionally, we observed a concurrent reduction in LA EF% and increase in LV ESV in HF‐CsA animals. LV end systolic/diastolic pressures (LV ESP and LV EDP, respectively) and peripheral MAP (distal to the aortic band) was significantly increased in HF‐CsA compared to HF and CON groups. The elevation of central and peripheral pressures in HF‐CsA animals suggests CsA treatment may have caused a hypertensive reaction. Heart rate was reduced in both HF and HF‐CsA groups, despite pressure being significantly elevated in HF‐CsA animals only.

### Diastolic mechanics

#### Late diastole

**Figure 2.**
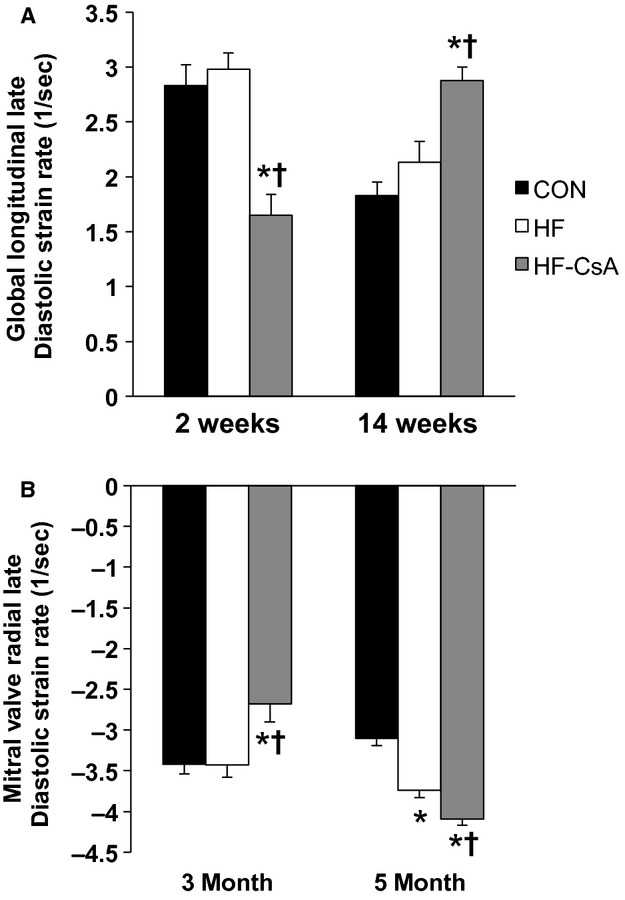
LV late diastolic longitudinal and mitral radial strain rates. (A–B) Cyclosporine acutely (2 weeks) reduces global late diastolic longitudinal and mitral valve radial strain rates, but increases both following 14 weeks of treatment. (*P < 0.05 vs. CON; ^†^*P* < 0.05 vs. HF)

The strain rate data presented in Figure 2 indicate CsA treatment acutely altered LV mechanics associated with atrial systole. Global longitudinal (Fig. 2A) and radial (at the level of the mitral valve; Fig 2B) late diastolic strain rates were reduced in HF‐CsA compared to CON and HF groups 2 weeks post–treatment. This effect was abrogated with chronic CsA treatment (14 weeks), as late diastolic strain rates were increased beyond that seen in the HF group. The measured values and increase in global mitral valve radial late diastolic strain rate observed in HF animals at 14 weeks are similar to previously published work by our laboratory (Marshall et al. 2013), supporting earlier observations of enhanced atrial systole in this HFpEF model.

#### Early diastole

**Figure 3.**
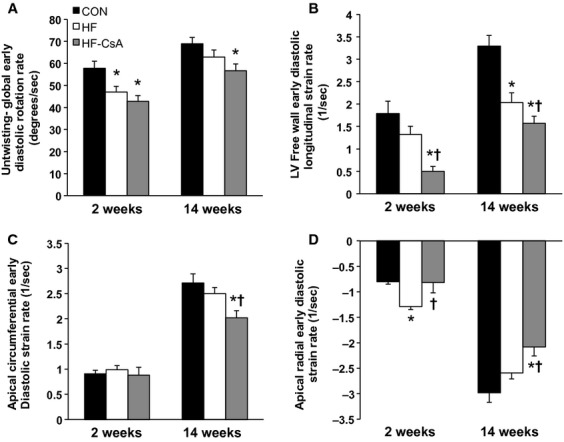
LV early diastolic untwisting and longitudinal, circumferential, and radial strain rates. (A) Peak global LV rotation rate during early diastole is reduced in both HF and HF‐CsA animals. (B) Longitudinal strain rate in the LV free wall during early diastole is reduced in following CsA treatment beyond that observed in HF animals. (C) Early diastolic circumferential strain rate in the apex of the LV is reduced after 14 weeks of CsA treatment. (D) Apical LV early diastolic radial strain rate is increased in HF animals 6 weeks post aortic banding (2 weeks), and reduced after 14 weeks of CsA treatment. (*P < 0.05 vs. CON; ^†^*P* < 0.05 vs. HF)

Acute and chronic treatment with CsA impaired LV mechanics associated with early LV diastolic filling beyond that observed in HF animals. Global LV untwisting rate during early diastole was reduced in HF‐CsA animals at 2 and 14 weeks compared to CON (Fig. 3A). A reduction in the rate of LV apical untwisting was associated with significant reductions in LV free wall longitudinal (Fig. 3B) and apical circumferential (Fig. 3C) and radial (Fig. 3D) strain rates. In HF animals, LV untwisting and longitudinal strain rate were reduced and apical radial/circumferential strain rates unchanged compared to CON animals, similar to findings previously reported by our lab (Marshall et al. 2013).

### Systolic mechanics

**Figure 4.**
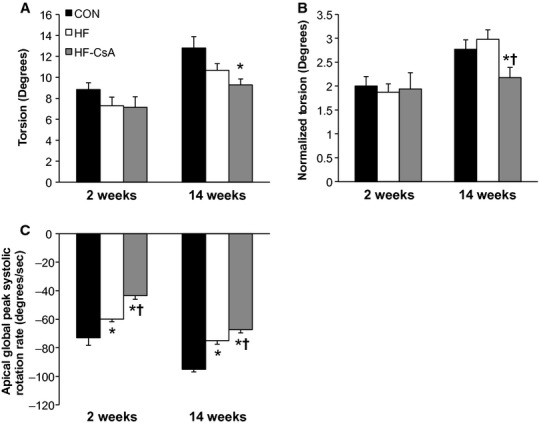
LV torsion and apical systolic rotation rates. (A–B) Torsion is reduced in HF‐CsA animals independent of normalization to LV morphology after 14 weeks of treatment. Preservation of torsion in the HF group recapitulates previous findings from our lab. (C) cyclosporine treatment reduces apical global peak systolic rotation rate beyond that observed in HF animals. (*P < 0.05 vs. CON; ^†^*P* < 0.05 vs. HF)

**Figure 5.**
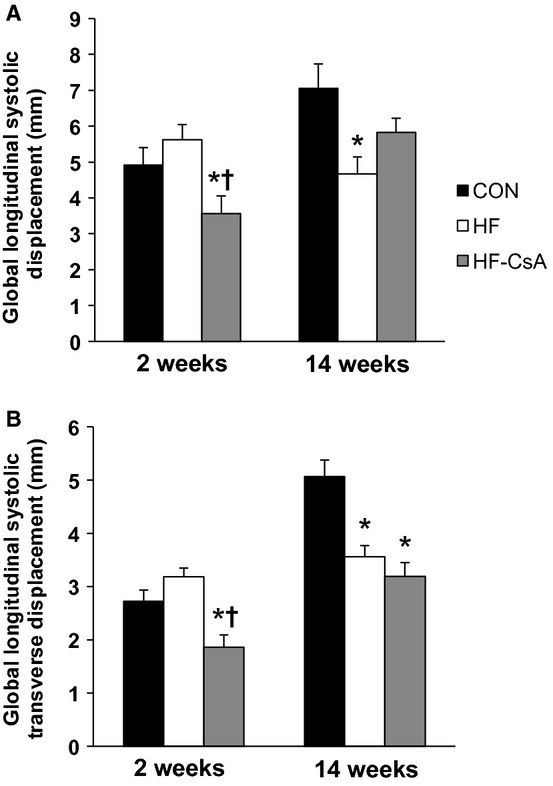
LV systolic longitudinal displacement. (A–B) Cyclosporine treatment reduces longitudinal (apex to base) and transverse longitudinal (free wall to septum) displacement during systole at 3 of 4 measured time points in contrast to HF animals, in which displacement was only reduced 20 weeks post aortic banding (14 weeks). (*P < 0.05 vs. CON; ^†^*P* < 0.05 vs. HF)

**Figure 6.**
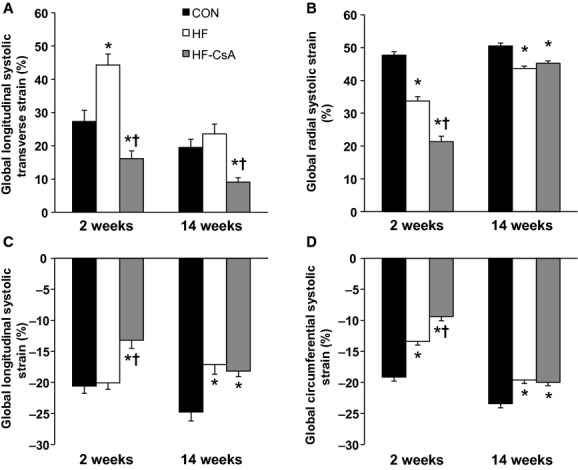
LV systolic longitudinal, radial, and circumferential strain. (A) Cyclosporine treatment reduces longitudinal transverse (free wall to septum) strain. (B) Radial systolic strain is acutely reduced in HF‐CsA animals beyond that observed in the HF group, but this difference is abrogated after 14 weeks of CsA treatment. (C) Cyclosporine treatment reduces longitudinal (apex to base) systolic strain at all measured time points in contrast to HF animals, in which displacement was only reduced 20 weeks post aortic banding (14 weeks). (D) Circumferential systolic strain is reduced in both HF‐CsA and HF animals compared to CON. (*P < 0.05 vs. CON; ^†^*P* < 0.05 vs. HF)

Treatment with CsA altered LV mechanics in a manner associated with diminished systolic function. LV torsion (absolute and normalized to LV wall thickness and end diastolic chamber length) was reduced in HF‐CsA animals after 14 weeks of treatment (Fig. 4A and B). Apical global peak systolic rotation rate (Fig. 4C) was reduced in HF animals compared to CON similar to our previously published findings (Marshall et al. 2013), and this effect was further exacerbated in the HF‐CsA group at both time points. Figure 5 shows decreased rotational movement in HF‐CsA animals was generally associated with concurrent reductions in global systolic longitudinal (5A) and longitudinal transverse (5B) displacement observed from an apical four‐chamber view. Strain measures presented in Figure 6 complement these findings, evident from reduced global longitudinal (6A), radial (6B), longitudinal transverse (6C), and circumferential (6D) systolic strain at all time points in the HF‐CsA group compared to CON. Reduced displacement in the longitudinal view was observed only at 14 weeks in HF animals (Fig. 5A and B). Global longitudinal transverse systolic strain (Fig. 6A) was not reduced in HF animals, and longitudinal transverse strain (Fig. 6C) was only decreased at 14 weeks. Global radial (Fig. 6B) and circumferential (Fig. 6D) systolic strain were decreased at all measured time points in the HF group compared to CON, but to a lesser degree than that observed in in HF‐CsA animals following 2 weeks of CsA treatment.

## Discussion

In this study, we provide a thorough examination of myocardial function following a novel drug intervention using a comprehensive combination of techniques including CT, 2D speckle tracking, and invasive hemodynamics in a translational large animal model of HFpEF. In the presence of existing hypertrophy and developing heart failure, our results indicate: (1) our novel CsA dosing scheme accelerated the development of early evidence for heart failure, including dilatory LV remodeling and impaired systolic and diastolic mechanics; (2) 2D speckle tracking was effective in differentiating between progressive gradations of developing heart failure; and (3) 2D speckle tracking is able to detect impaired diastolic LV mechanics early in the disease process prior to the development of overt diastolic dysfunction.

It is well established that conventional therapies proven effective in HFrEF have failed to improve the prognosis of HFpEF patients over the past three decades, leading to an unacceptably high rate of mortality and illustrating the need for the development of novel therapeutic strategies (Maeder and Kaye 2009; Paulus and van Ballegoij 2010; Borlaug and Paulus 2011; Borlaug and Redfield 2011; Burkhoff 2012). The CsA dosage administered in this study was based on the effective dose given to humans in an early clinical trial for ischemia‐reperfusion/myocardial infarction treatment (Piot et al. 2008), which has now moved into a phase III clinical trial (The CIRCUS trial). A low dose of CsA (1 mg kg^−1^ day^−1^) was shown to inhibit the MPT pore and beneficially influence cardiac remodeling in mice (Marechal et al. 2011), and 2 mg kg^−1^ day^−1^ is 5–10 times lower than the dose of CsA (10‐20 mg kg^−1^ day^−1^) used for immunosuppression in the pig (Okumi et al. 2008; Rigol et al. 2008). Thus, the study was designed to prevent inhibition of calcineurin and/or subsequent immunosuppression focusing solely on inhibition of cyclophilins and related MPT. We hypothesized this novel twist on a well‐known drug could improve myocardial energetics independent of its classic use as an inhibitor of myocardial remodeling, thus improving diastolic mechanics and function in HFpEF.

In contrast to this objective, the most interesting finding of the study was that chronic treatment with low doses of CsA induced LV dilation and exacerbated systolic and diastolic dysfunction during developing heart failure, refuting our initial hypothesis. Although our results indicate the novel CsA dosing scheme administered in this study does not appear to be an effective therapeutic alternative for HFpEF, the accelerated development of heart failure in treated animals provided a unique opportunity to evaluate 2D speckle tracking as an effective means of identifying distinct stages of LV function along a spectrum of developing heart failure. CsA‐dependent dilatory remodeling of the LV was associated with altered LV mechanics associated with reduced systolic emptying, impaired early diastolic filling, and enhanced atrial systole that extended beyond that observed in HF animals. These mechanical measures were associated with impaired hemodynamics and cardiac function. Specifically, reductions in cardiac torsion (Fig. 4A and B), apical systolic rotation rate (Fig. 4C), and systolic strain values (Fig. 6) were reflected in declining LV systolic function (EF = 47%) and increased LV ESV and ESP (Table 1). Reductions in early diastolic untwisting (Fig. 3A) and strain rates (Fig. 3B–D) reflect diastolic dysfunction evident as an increase in LV EDP and EDV (Table 1). Previous clinical studies have demonstrated the gradual decline of torsion, twisting, and strain measures with the progressive development of heart failure (Takeuchi et al. 2007; Kosmala et al. 2008; Park et al. 2008), and the depression of LV mechanical measures associated with systolic and early diastolic dysfunction measured following 14 weeks of treatment in the HF‐CsA group matches these observations. In summary, our 2D speckle tracking data display excellent coherence with our hemodynamic and LV functional data, suggesting dilation of the LV is associated with a parallel and comprehensive deterioration of normal mechanical function.

Augmenting atrial systole is another common compensatory mechanism of preserving diastolic filling in heart failure patients, and evidence of this is present in both the HF and HF‐CsA groups and reflected as an increase in late diastolic longitudinal (Fig. 2A) and radial (Fig. 2A) strain rate at 14 weeks. Interestingly, 2 weeks of CsA treatment acutely reduced both of these measures suggesting the contributions of atrial systole to LV filling during late diastole was reduced. This type of mechanical change in the context of HFpEF, in which diastolic function and LV filling is typically impaired, would be considered a positive adaptation. However, over the course of treatment these values increased to a greater degree in HF‐CsA animals and coincided with a significant increase in LV EDP. These findings suggest this common compensatory mechanism of preserving diastolic filling in HF patients, observed in this study and previously in our HFpEF swine model (Marshall et al. 2013), was made worse by chronic CsA treatment. The differential response to acute and chronic CsA treatment is difficult to reconcile, however, we speculate it could be the result of LV remodeling. The concentric hypertrophy evident in HF‐CsA animals (as indicated by increased LV diastolic wall thickness) at 2 weeks of CsA treatment when compared to the dilatory LV remodeling displayed after completing our dosing regimen suggests significant changes in wall stress could exist and thus, have substantial subsequent impact on LV mechanics. Although our findings suggest an acute, low dose of CsA may attenuate enhanced late diastolic mechanics commonly observed in HFpEF, the clinical relevance of these data is questionable when compared to our summary findings of globally impaired LV mechanical function following chronic CsA treatment.

Interestingly, LV mechanics associated with systolic dysfunction was also observed in the HF group. These findings appear counterintuitive, as HFpEF patients typically demonstrate preserved systolic function at rest. Preservation of resting systolic function is a common feature of HFpEF, although decreased cardiac functional reserve is well established in this patient population (Tan et al. 2009; Norman et al. 2011). The coexistence of diagnostic indices of both normal and reduced systolic function is common and difficult to reconcile in HFpEF, thus, it remains controversial whether LV systolic function remains normal in these patients. Recently published data from our lab demonstrate that this paradox exists in our model (Marshall et al. 2013) and indeed, results from this study show that depressed systolic mechanics was observed with other markers of preserved systolic function including maintenance of normal EF% (>50%) and LV end systolic volumes in the HF group. Our findings suggest that 2D speckle tracking may be an effective and sensitive means of uncovering early systolic dysfunction in HFpEF patients before the deterioration of other more common markers of systolic function, such as EF%, are observed. Although the clinical implications of our findings are yet to be determined, the divergent nature of LV mechanics observed between the three experimental groups in this study suggests 2D speckle tracking has diagnostic and therapeutic significance that could potentially improve our understanding of the development and treatment of HFpEF.

Recently, 2D speckle tracking echocardiography has received increasing attention as a clinically relevant diagnostic tool for assessing myocardial function in HFpEF patients (Kosmala et al. 2008; Norman et al. 2011; Yip et al. 2011; Morris et al. 2012a). Adding to this body of literature, our results suggest 2D speckle tracking is able to detect altered LV mechanics associated with impaired systolic and diastolic function prior to the onset of overt diastolic dysfunction evident as increased LV end diastolic pressure. These data parallel recent published results from our laboratory which demonstrated diastolic dysfunction and impaired contractile reserve, hallmark features of HFpEF, were present without a significant increase in LV end diastolic pressure (≤15 mmHg) in animals exhibiting EF > 50% (Marshall et al. 2013). This finding carries clinical significance, as recent evidence from (Morris et al. 2012a) suggests alterations to LV mechanics associated with impaired systolic and diastolic performance in HFpEF are linked to increases in LV filling pressure. Clinically, LV filling pressure is commonly assessed noninvasively using the mitral E/e' ratio. An E/e' ratio of >15 is indicative of elevated LV filling pressures, and this standard is used as a key component of the diagnostic determinants for the evaluation of HFpEF (Paulus et al. 2007; Nagueh et al. 2009). Clinical studies examining 2D speckle tracking and/or LV filling pressures in HFpEF typically include NYHA class ≥2 patients in which filling pressures are already elevated (Borlaug et al. 2010a; Maeder et al. 2010; Norman et al. 2011; Yip et al. 2011; Morris et al. 2012a), although recent evidence suggests E/e' ratios are not always elevated in these patients (Kosmala et al. 2008; Phan et al. 2009; Tan et al. 2009; Borlaug et al. 2010a; Maeder et al. 2010; Norman et al. 2011). Indeed, several studies performed in HFpEF patients report mitral E/e' ratios of <15, with many reporting values <10 (Kosmala et al. 2008; Tan et al. 2009; Borlaug et al. 2010a; Maeder et al. 2010; Norman et al. 2011). In total, our results suggest that LV diastolic mechanics may be impaired prior to increases in LV end diastolic pressure and 2D speckle tracking could potentially enhance earlier detection of diastolic dysfunction in patients at risk for developing HFpEF.

Finally, we addressed the possibility that data generated in the current and previous studies by our lab were unique to the animal model. Systolic longitudinal strain and strain rate values in CON animals were similar to recently reported norms reported for healthy adults (Lotfi‐Tokaldany et al. 2013), and we previously demonstrated torsion and untwisting rates correlate to traditional indices such as EF%, LV ESV, and Tau (Marshall et al. 2013) as previously observed in humans (Dong et al. 2001; Wang et al. 2007; Notomi et al. 2008). Torsion and longitudinal, circumferential, and radial strain measurements in all animals from this study were also directly comparable to published human values (Wang et al. 2007; Kosmala et al. 2008; Park et al. 2008; Phan et al. 2009; Tan et al. 2009; Yip et al. 2011; Morris et al. 2012a,b). Furthermore, data in this study showing preserved torsion, diminished apical systolic and early diastolic rotation rates, and enhanced late diastolic radial strain in the HF group recapitulate previous results from our lab (Marshall et al. 2013). Although these findings do not formally establish reproducibility, our confirmation of similar outcome measures from two different studies, two different sets of animals, and two different analysts importantly demonstrates the consistency of the model and supports its relevance clinically as a model of HFpEF.

Although outside the hemodynamic and imaging focus of this study, we are currently examining multiple molecular mechanisms regarding the detrimental cardiac outcomes observed following our CsA dosing regimen. Regarding systemic hemodynamic mechanisms, it is well known that CsA can cause hypertension (Hoorn et al. 2012), although the dose used in this study (on the low end of the dosing spectrum) has only been associated with increases in mean blood pressure of 5 mmHg on average (Robert et al. 2010). In this context, it is possible the increased systolic, diastolic, and MAPs observed in the HF‐CsA group accelerated the progression to LV dilation in the face of our sustained increase in afterload as a result of chronic aortic‐banding, representing a functional systemic mechanism underlying our findings. Endothelin‐1 (ET‐1) is known to contribute to the renal hypertension observed following treatment with calcineurin inhibitors such as CsA (Perico et al. 1990; Lanese and Conger 1993; Textor et al. 1995; Cavarape et al. 1998), and previous studies in rats have demonstrated ET‐1 plays a role in CsA‐induced hypertension (Takeda et al. 1995). However, it is still unclear if the effects of ET‐1 on calcineurin inhibitor‐dependent hypertension are the result of systemic or renal influences. We have previously shown enhanced coronary vascular sensitivity to ET‐1 in our swine HFpEF model (Emter et al. 2011) and, although speculative, a parallel adaptation in the peripheral vasculature could be a systemic mechanism fundamental to the hypertension and related dilatory LV remodeling observed in this study. Furthermore, calcineurin inhibitor‐dependent hypertension has also been linked to sodium retention (Curtis et al. 1988). Subsequent increases in systemic fluid volume may have also played a role in both the hypertensive response and significant increase in weight observed in the HF‐CsA group.

### Limitations

This study used a comprehensive combination of techniques including CT, 2D speckle tracking, and invasive hemodynamics difficult to duplicate clinically, however, there are limitations. A recent editorial addressed the topic of variability in strain measures regarding why 2D speckle tracking has not become standard for clinical diagnosis of heart disease (Marwick 2012). A growing amount of evidence exists regarding the considerable intra‐ and intervariability observed in strain measures (Oxborough et al. 2012; Risum et al. 2012). Strain variability has been shown to be directionally dependent, with radial and transverse strain showing the highest variability and coefficients of variation ranging from 6 to 33% (Marwick 2012; Risum et al. 2012). Furthermore, variability in strain rate is greater compared to strain, and substantial variability exists when moving from apical to basal segments (Leung and Ng 2010; Oxborough et al. 2012). In this study, a great deal of variation is observed in CON animal strain rates at our 2 and 14 week time points. We believe this to be in part both methodological and as a result of normal physiological growth. Strain measures can be impacted by the complexity of myocardial fiber orientations, resulting in the capture of different fiber layers at multiple levels and variable results (Bauer et al. 201; Risum et al. 2012). In this regard, CON animals in this study showed normal physiological growth evident as an increase in mean weight from 27 to 35 kg. We have previously published this growth is reflected in both animal weight and an increase in cardiac size (Emter and Baines 2010). Changes in overall body size and physiological heart growth can lead to alterations in beam direction and potentially the capture of different myocardial layers or fiber orientation, ultimately leading to changes in the number and/or size of speckle kernels for analysis (Risum et al. 2012). The studies outlined above provide insight regarding the variability in CON animal strain rates observed herein, and demonstrate the need for continuing research regarding the routine implementation of 2D speckle tracking for clinical diagnosis of heart disease.

In conclusion, our results indicate chronic low‐dose CsA treatment accelerates the development of heart failure. Although our findings do not support the use of low‐dose CsA as a viable therapeutic alternative for HFpEF, our results demonstrate 2D speckle tracking is effective in differentiating between progressive gradations of developing heart failure. Furthermore, we were able to detect impaired diastolic LV mechanics prior to the development of overt diastolic dysfunction. Our findings add to the growing body of evidence suggesting 2D speckle tracking holds significant potential as a clinically relevant diagnostic tool for assessing myocardial function in heart failure patients.

## Acknowledgments

The authors thank Melissa Cobb, Jan Ivey, and Kurt Marshall for their considerable technical contributions, which were essential to the successful completion of the study.

## Conflict of Interest

None declared.
